# RCS–Doppler-Assisted MM-GM-PHD Filter for Passive Radar in Non-Uniform Clutter

**DOI:** 10.3390/s25185864

**Published:** 2025-09-19

**Authors:** Jia Wang, Baoxiong Xu, Zhenkai Zhang, Biao Jin

**Affiliations:** Ocean College, Jiangsu University of Science and Technology, Zhenjiang 212100, China; 232241803426@stu.just.edu.cn (J.W.); zhangzhenkai@just.edu.cn (Z.Z.); biaojin@just.edu.cn (B.J.)

**Keywords:** target tracking, multiple model probability hypothesis density, feature matching, missed alarm, non-uniform clutter

## Abstract

In passive radar, the multiple model probability hypothesis density (MM-PHD) filter has demonstrated robust capability in tracking multi-maneuvering targets. Nevertheless, non-uniform clutter in practical scenarios causes misestimation of component weights, thereby generating false targets. To solve the false targets problem, a feature-matching MM-PHD (FM-MM-GM-PHD) algorithm for passive radar tracking is proposed in this paper. First, the measurement likelihood function was refined by leveraging target radar cross-section (RCS) and Doppler features to assist in suppressing false targets and reduce clutter interference. Additionally, the proposed algorithm incorporated adaptive component pruning and absorption processes to enhance tracking accuracy. Finally, a missed-alarm correction mechanism was introduced to compensate for measurement losses. Simulations of the passive radar results validated the findings that the proposed algorithm outperformed the traditional MM-PHD filter in both tracking accuracy and cardinality estimation. This superiority was particularly pronounced in non-uniform clutter environments under low detection probabilities.

## 1. Introduction

Traditional active radar suffers from inherent limitations. Its radiated signals are easily intercepted, and it offers weak anti-interference capability. In complex environments, this radar’s performance faces severe restrictions. Passive radar uses external signals, and this approach offers distinct advantages, including outstanding concealment and low cost. Research on target tracking algorithms for passive radar holds significant value in the complex electromagnetic environment.

In the field of passive radar tracking, multi-target tracking has always been a highly challenging problem [1]. The complexity of this problem stems from the need to evaluate the motion characteristics of multiple targets simultaneously based on measurements. The uncertainty of target motion and the variation in the number of targets further compound these intractable tracking difficulties.

Conventional multi-target tracking (MTT) approaches rely on data-association techniques [2,3,4], including multiple hypothesis tracking (MHT), joint probability data association (JPDA), and probability data association (PDA) [5,6]. Such methods break down the MTT challenge into individual single-target problems, addressing the task of assigning measurements to targets. Nevertheless, their complexity increases sharply as the number of measurements increases. Over recent years, MTT methods based on random finite sets (RFS) have received widespread attention since they can naturally handle changes in the number of targets and measurement uncertainty [7,8,9]. Some algorithms such as probability hypothesis density (PHD) [10,11,12], cardinalized probability hypothesis density (CPHD) [13,14,15], and generalized labeled multi-Bernoulli (GLMB) [16,17,18] can directly estimate the state, number, and trajectory information of multiple targets without data association. PHD and CPHD are widely used in engineering due to their low implementation difficulty and low computational complexity.

The above-mentioned RFS algorithms mainly focus on tracking targets using a single motion model. However, a single model is insufficient to effectively track maneuvering targets, necessitating the use of multiple models to modify their motion [19]. The interacting multiple model (IMM) method was introduced in [20,21] to sacrifice complexity for higher tracking accuracy. In [22,23], the ordinary multiple model (MM) method was presented to quickly track multiple maneuvering targets through sequential Monte Carlo (SMC) and Gaussian mixture (GM). While the SMC method has better tracking accuracy in non-linear problems, target number estimation with SMC degrades due to unreliable clustering operations. In engineering, the MM-PHD method is widely used in the form of GM solution for its high reliability.

In the modern complex electromagnetic environment, the distribution of clutter is often non-uniform. In uniform clutter environments, MM-GM-PHD effectively handles target motion changes. However, dense clutter in non-uniform clutter misleads the multiple model method. This generates false targets and weakens tracking performance. Dense clutter fills multiple model gates with false measurements and leads to severe misestimation of target number, states, and motion models. Existing non-uniform clutter suppression techniques at the radar data processing stage [24,25,26] apply to single-target motion model. These algorithms tend to inadequately address the limitations of the multiple model PHD method in non-uniform clutter. Feature-aided modification of the target likelihood function provides a promising approach to address these challenges. Tian et al. integrated direction-of-arrival (DOA) and frequency features to construct a joint position-feature likelihood function, improving measurement association [27]. Zheng et al. utilized RCS and Doppler characteristics to adjust measurement likelihood weights in space-based target tracking [28]. Yu and Xi-an employed spread angle measurements to reformulate the likelihood model for sonar extended targets in heterogeneous fusion [29]. Bai et al. designed an SNR-aware factor to modify the likelihood function against interference [30]. Wei et al. developed a hybrid likelihood model to track coexisting point and extended targets [31]. Inspired by the above methods, we propose a feature-matching MM-GM-PHD (FM-MM-GM-PHD) algorithm. It corrects measurement likelihood using target RCS and Doppler features. The filter effectively tracks maneuvering targets and solves the performance degradation problem of the multiple model PHD filter in non-uniform clutter.

Additionally, passive radar in complex environments is prone to miss alarms caused by external interference [32]. This significantly increases tracking difficulty. This paper proposes a missed-alarm correction mechanism. It can correct the estimation of target states according to historical multi-target states, thus reducing the impact of missed alarms on tracking.

The main contributions are as follows:

(1) For passive radar, we propose a feature-matching multiple model PHD tracking method that combines RCS and Doppler features. This method solves the issue of abnormal component weights of multiple models in non-uniform clutter environments which are likely to generate false targets.
(2) For non-uniform clutter environments, we improve the pruning and merging of Gaussian components and reduce the likelihood of mis-merging through adaptively adjusting the merging threshold based on Gaussian component weights.
(3) To tackle the issue of missed alarms concerning targets, we introduce a missed-alarm correction mechanism. By leveraging the variations in the target’s motion states within the prior two frames, this mechanism adaptively adjusts the predicted state.

## 2. Problem Description

Tracking multiple targets with passive radar is of great significance. In practical monitoring scenarios, clutter distributions are often non-uniform. This non-uniformity poses a great challenge to passive radar in tracking multiple targets.

### 2.1. Missed Alarm

**Figure 1:**
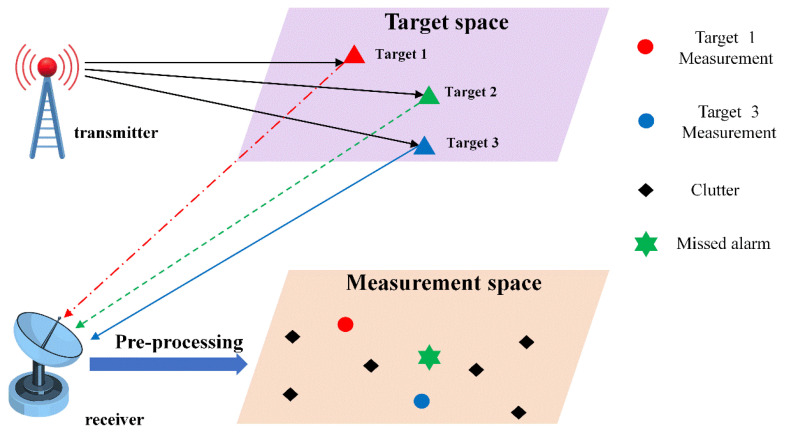
Schematic diagram of passive radar tracking.

As illustrated in Figure 1, passive radar systems face critical tracking challenges in non-uniform clutter environments. Natural and man-made interferences cause clutter distributions to exhibit strong spatial variations, with intensities near the receiver significantly exceeding target echoes. When local clutter overwhelms targets, the signal-to-clutter ratio (SNR) drops precipitously, leading to missed alarms (e.g., Target 2 in Figure 1, where no measurement is generated). Concurrently, false measurements arise from persistent clutter (e.g., the isolated “Clutter” point in Figure 1 measurement space). This directly induces tracking anomalies in Bayesian algorithms—target track disruption due to measurement loss, false track initiation from clutter points, and re-entry misdetections when targets revisit surveillance areas. The coexistence of undetected targets and false measurements challenges traditional frameworks in sustaining continuous state estimation under the complex clutter–echo interactions.

### 2.2. False Targets Caused by MM-PHD

**Figure 2:**
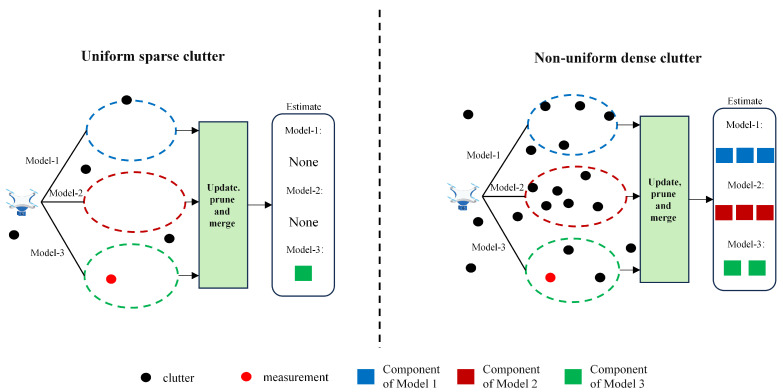
Schematic diagram of multi-model PHD tracking under different clutter distributions.

The multi-model approach is currently a mainstream method for maneuvering targets. To achieve better tracking performance, the model set should include as many models as possible that match the target’s motion and as few mismatched models as possible. However, this creates a contradiction in engineering practice. At a certain moment, the target must move in only one way, while other models in the model set will degrade the performance of the filter. The above phenomenon intensifies while targets enter a dense clutter environment. Dense clutter significantly affects the weight calculation process in multi-model approaches. Within this framework, some models mismatched with the target actual motion generate misleading feedback due to clutter interference. This interference causes the weight values of these mismatched model components to increase abnormally during calculation. Figure 2 illustrates this process, visually showing how clutter impacts component weight distribution.

It is not difficult to find that dense clutter increases the likelihood of false measurements falling into the prediction gates of mismatched motion models. This makes the component weights of the mismatched models increase, generating multiple false targets. It leads to obvious deviations in the estimation of the number and states of targets.

## 3. The Proposed Method

**Figure 3:**
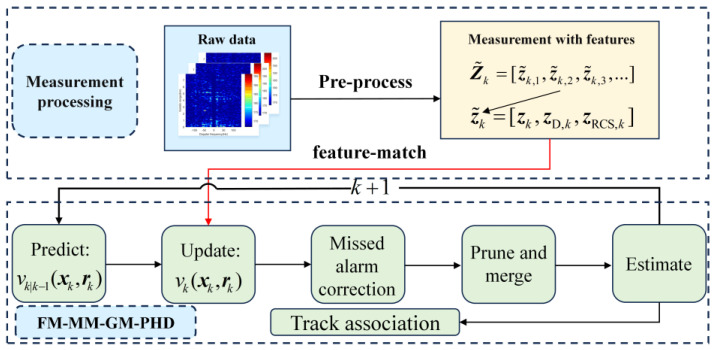
Flowchart of the proposed algorithm.

This section elaborates the prediction and update procedures of the FM-MM-GM-PHD filter, with its algorithmic flowchart presented in Figure 3.

### 3.1. PHD Filter

The PHD filter operation encompasses prediction and update phases. Let the predictive intensity and posterior intensity at time k be defined as $v_{k|k-1}$ and $v_{k}$, respectively. The formula can be defined as

(1) $$\begin{array}{l} v_{k|k-1}(x_{k})=\gamma_{k}(x)+\\ \;\;\;\;\;\;\;\;\;\;\;\;\;\;\;\;\;\;\int p_{s,k|k-1}(x_{k-1})f_{k|k-1}(x_{k}|x_{k-1})v_{k-1}(x_{k-1})dx_{k-1}+\\ \;\;\;\;\;\;\;\;\;\;\;\;\;\;\;\;\;\;\int \beta_{k|k-1}(x_{k}|x_{k-1})v_{k-1}(x_{k-1})dx_{k-1}, \end{array}$$

where $p_{s,k|k-1}(x_{k-1})$ denotes the survival probability of targets, and $f_{k|k-1}(\cdot |x_{k-1})$ signifies the state transition probability intensity. $\gamma_{k}$ denotes the birth intensity of targets at time k. $\beta_{k|k-1}(\cdot |x_{k-1})$ denotes the spawning probability intensity. In the subsequent content of this article, we do not consider the spawning processes.

After prediction, the PHD update step can be given by

(2) $$\begin{array}{l} v_{k}(x_{k})=[1-p_{D,k}(x_{k})]v_{k|k-1}(x_{k})+\\ \;\;\;\;\;\;\;\;\;\;\;\;\;\;\;\sum_{z\in Z_{k}}\frac{p_{D,K}(x_{k})g_{k}(z|x_{k})v_{k|k-1}(x_{k})}{K_{k}(z)+\int p_{D,K}(\eta_{k})g_{k}(z|\eta_{k})v_{k|k-1}(\eta_{k})d\eta_{k}}, \end{array}$$

where $p_{D,k}(x_{k})$ signifies the target detection probability at time k, $g_{k}(\cdot |x_{k})$ denotes the measurement likelihood function of the target at time step k. $K_{k}(\cdot )$ represents the clutter intensity.

Notably, the conventional implementation of PHD filter incorporates numerous assumptions, such as unchanging clutter intensity and invariant detection probability. The recursion at time k is underpinned by the following premises:

- The observation data is modeled as an RFS composed of valid measurements from targets and background clutter. Target observations and clutter are assumed to be mutually independent.
- Clutter is hypothesized to follow a Poisson distribution, with its intensity treated as a known constant.
- The detection procedure for each target is mutually independent, with a fixed detection probability.

### 3.2. FM-MM-GM-PHD

Within this subsection, the proposed algorithm undergoes elaborate derivation, with an analytical implementation approach presented. This method leverages target RCS and Doppler signatures to facilitate tracking multiple maneuvering targets.

#### 3.2.1. RCS and Doppler Measurement Modeling

In practical monitoring environment, non-uniform clutter is mainly caused by ground objects, meteorological conditions, and other non-target objects. Ground clutter results from the scattering of radar waves by ground objects. It has high intensity and dense distribution. The RCS of ground clutter is typically several times larger than the target RCS. Since ground objects are stationary, the Doppler frequency of the clutter is basically zero. Meteorological clutter is caused by the scattering of radar waves by meteorological particles such as rain, snow, and fog. Such clutter has weak intensity and a more dispersed distribution. Considering these clutter features, this paper uses RCS and Doppler frequency features to assist tracking.

The pre-processed target measurements include state information and Doppler frequency and RCS features. The measurement state of targets at time k is defined as

(3) $$z_{k}=[z_{L,k},z_{D,k},z_{\mathrm{RCS},k}],$$

where $z_{L,k}$ denotes the target position measurement at time step k, while $z_{D,k}$ and $z_{\mathrm{RCS},k}$ represent its Doppler and RCS measurements, respectively.

Under the assumption that target Doppler information and RCS are independent of its position, the likelihood models for the target and clutter are formulated as

(4) $$g(z|x)=g_{z}(z|x)g_{D}(D)g_{R}(RCS),$$

(5) $$s(z|x)=s_{z}(z|x)s_{D}(D)s_{R}(RCS),$$

where $g_{z}(z|x)$, $g_{D}(D)$, and $g_{R}(RCS)$ are the likelihoods of the target position, Doppler frequency, and RCS, respectively. $s_{z}(z|x)$, $s_{D}(D)$, and $s_{R}(RCS)$ are the likelihood functions of the clutter regarding position, Doppler frequency, and RCS, respectively.

Specifically, under the assumption that these two quantities are conditionally independent of the target’s position given the target state vector, this relationship holds because the Doppler shift is determined by the radial velocity, whereas the RCS depends on the target’s physical properties. Since both the radial velocity and physical properties are inherently encoded within the target state vector, this conditional independence assumption is well-founded. The likelihood function of the Doppler frequency can be expressed as

(6) $$g_{D}(D)=N(z_{D,k},h_{k}(x_{k}),R_{D,k}),$$

(7) $$h_{k}(x_{k})=x_{k}{\dot{x}}_{k}/\sqrt{x_{k}^{2}+y_{k}^{2}}+y_{k}{\dot{y}}_{k}/\sqrt{x_{k}^{2}+y_{k}^{2}},$$

where $R_{D,k}$ denotes the noise covariance matrix, $x_{k}$, $y_{k}$ along with ${\dot{x}}_{k}$; ${\dot{y}}_{k}$ describe the target state in the x–y plane of the Cartesian coordinate system at time step k.

Target RCS follows a chi-squared distribution model, with its likelihood function defined as

(8) $$g_{\mathrm{RCS}}(RCS)=\frac{m}{\Gamma (m)RCS_{\mathrm{av}}}{\left(\frac{mRCS}{RCS_{\mathrm{av}}}\right)}^{m-1}e^{\frac{-mRCS}{RCS_{\mathrm{av}}}},$$

where $\Gamma (m)=\int_{0}^{\infty }t^{m-1}e^{-t}dt$ denotes the gamma function, m represents the degrees of freedom, and $RCS_{\mathrm{av}}$ signifies the target average RCS value.

It should be noted that the chi-squared distribution assumption for RCS in this method is applicable to point targets with a single dominant scattering center. For such targets, the radar echo amplitude follows a complex Gaussian distribution, and the squared magnitude of the echo amplitude conforms to the chi-squared model, thereby ensuring the validity of the established RCS likelihood function. Minor deviations from the chi-squared distribution can be partially compensated for by the orthogonality between RCS and Doppler features within the feature-matching framework. Regarding target adaptability, the current method is designed for point targets, aligning with typical requirements for point-target tracking in passive radar systems. Extended targets, on the other hand, would require additional modeling of their spatial extent and surface multi-scattering characteristics, which falls outside the scope of this study. While the proposed multi-model feature fusion framework may support future extensions to handle extended targets, this is not the focus of the present work.

#### 3.2.2. Prediction

For a filter implemented with GM, if the posterior intensity at time k−1 is given by

(9) $$v_{k-1}(x_{k-1},r_{k-1})=\sum_{j=1}^{J_{k-1}(r_{k-1})}\omega_{j,r_{k-1}}^{k-1}N(x;m_{j,r_{k-1}}^{k-1},P_{j,r_{k-1}}^{k-1}),$$

the predicted intensity $v_{k|k-1}(x_{k},r_{k})$ can be expressed as

(10) $$v_{k|k-1}(x_{k},r_{k})=v_{f,k|k-1}(x_{k},r_{k})+v_{\gamma ,k}(x_{k},r_{k}),$$

$v_{f,k|k-1}(x_{k},r_{k})$ donates the survival intensity and has the following form

(11) $$v_{f,k|k-1}(x_{k},r_{k})=\sum_{r_{k-1}}\sum_{j=1}^{J_{k-1}(r_{k-1})}\omega_{f,k|k-1}^{j}(r_{k},r_{k-1})M(r_{k},r_{k-1}),$$

where

(12) $$M(r_{k},r_{k-1})=N(x;m_{f,k|k-1}^{j}(r_{k},r_{k-1}),P_{f,k|k-1}^{j}(r_{k},r_{k-1}))$$

(13) $$\omega_{f,k|k-1}^{j}(r_{k},r_{k-1})=p_{s,k|k-1}(r_{k-1})t_{k|k-1}(r_{k}|r_{k-1})\omega_{k-1}^{j}(r_{k-1}),$$

(14) $$m_{f,k|k-1}^{j}(r_{k},r_{k-1})=F_{k-1}(r_{k})m_{k-1}^{j}(r_{k-1}),$$

(15) $$P_{f,k|k-1}^{i}(r_{k},r_{k-1})=Q_{k-1}(r_{k})+F_{k-1}(r_{k})P_{k-1}^{i}(r_{k-1}){\left[F_{k-1}(r_{k})\right]}^{T},$$

$p_{s,k|k-1}(r_{k-1})$ denotes the target survival probability under model $r_{k-1}$, $t_{k|k-1}(r_{k}|r_{k-1})$ denotes the motion model transition probability from $r_{k-1}$ to $r_{k}$, and $\omega_{k-1}^{j}(r_{k-1})$ denotes the weight of the j-th component under model $r_{k-1}$.

#### 3.2.3. Update

In the FM-MM-GM-PHD filter, the predicted intensity in GM form $v_{k|k-1}(x_{k},r_{k})$ is formulated as

(16) $$v_{k|k-1}(x_{k},r_{k})=\sum_{j=1}^{J_{k|k-1}(r_{k})}\omega_{j,r_{k}}^{k|k-1}N(x;m_{j,r_{k}}^{k|k-1},P_{j,r_{k}}^{k|k-1}),$$

the posterior intensity is hereby expressed as

(17) $$v_{k}(x_{k},r_{k})=[1-P_{D,k}(x)]v_{k|k-1}(x_{k},r_{k})+v_{k|k-1}^{Z_{k}}(x_{k},r_{k}),$$

where

(18) $$v_{k|k-1}^{Z_{k}}(x_{k},r_{k})=\sum_{z\in Z_{k}}\sum_{j=1}^{J_{k|k-1}(r_{k})}\omega_{j,D,r_{k}}^{k}N(x;m_{j,r_{k}}^{k}(z),P_{j,r_{k}}^{k}),$$

(19) $$\omega_{j,D,r_{k}}^{k}=\frac{A_{j,D,r_{k}}^{k}(x)\omega_{j,r_{k}}^{k|k-1}q_{j,r_{k}}(z)}{\lambda_{c}c(z)s_{R}(RCS)s_{D,k}(D{)+G}_{j,D,r_{k}}^{k}(z)},$$

(20) $$G_{j,D,r_{k}}^{k}(z)=\sum_{r_{k}}A_{j,D,r_{k}}^{k}(x)\sum_{l=1}^{J_{k|k-1}(r_{k})}\omega_{l,r_{k}}^{k|k-1}q_{l}(z),$$

(21) $$A_{j,D,r_{k}}^{k}(x)=P_{D,k}(x)g_{z}(z|x)g_{R}(RCS)g_{D,k}(D),$$

(22) $${\tilde{z}}_{j,r_{k}}=H_{r_{k}}^{k}m_{j,r_{k}}^{k|k-1},$$

(23) $$q_{j,r_{k}}(z)=N(z;{\tilde{z}}_{j,r_{k}},H_{r_{k}}^{k}P_{j,r_{k}}^{k|k-1}{\left[H_{r_{k}}^{k}\right]}^{T}+R_{j,r_{k}}^{k}),$$

(24) $$m_{j,r_{k}}^{k}=m_{j,r_{k}}^{k|k-1}+K_{j,r_{k}}^{k|k-1}(z-{\tilde{z}}_{j,r_{k}}),$$

(25) $$P_{j,r_{k}}^{k}=P_{j,r_{k}}^{k|k-1}-K_{j,r_{k}}^{k}H_{r_{k}}^{k}P_{j,r_{k}}^{k|k-1},$$

(26) $$K_{j,r_{k}}^{k}=P_{j,r_{k}}^{k|k-1}{\left[H_{r_{k}}^{k}\right]}^{T}\cdot {\left(H_{r_{k}}^{k}P_{j,r_{k}}^{k|k-1}{\left[H_{r_{k}}^{k}\right]}^{T}+R_{r_{k}}^{k}\right)}^{-1}.$$

Here, $\lambda_{c}c(z)$ denotes the clutter intensity, $s_{R}(RCS)$ and $s_{D,k}(D)$ denote the likelihood functions of the clutter RCS and Doppler frequency. The likelihood function $q_{j,r_{k}}(z)$ represents the measurement likelihood function of the j-th component under the $r_{k}$. $K_{j,r_{k}}^{k}$ denotes the Kalman gain of the j-th component under the $r_{k}$, $H_{r_{k}}^{k}$ denotes the observation matrix under the $r_{k}$, and $R_{r_{k}}^{k}$ denotes the measurement noise covariance matrix under $r_{k}$.

The expectation of the target number is

(27) $$E(N_{k})=\int v_{k}(x)dx,$$

where E(⋅) denotes the expectation symbol. For the standard PHD update Equation (2), in our method, $g_{k}(z|x_{k})$ is replaced by joint feature-state likelihood $g_{z}(z|x)g_{R}(RCS)g_{D,k}(D)$ and $\int g_{z}(z|x)g_{R}(RCS)g_{D,k}(D)=1$, the modified update retains the PHD form. This proves that the first-moment property is preserved.

#### 3.2.4. Judgment of Missed Alarm

**Figure 4:**
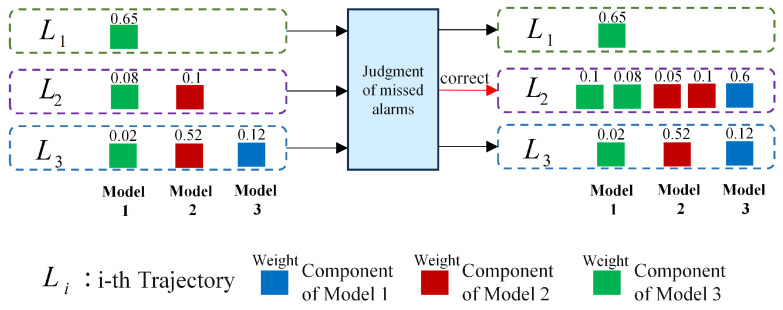
Schematic diagram of missed-alarm correction algorithm.

A missed alarm is determined based on two key conditions, as visualized in Figure 4. First, the weight of this dominant component must be lower than a predefined threshold. This threshold is set to reflect the minimum weight required to confirm the presence of valid measurements, and its value is adjusted according to clutter intensity to avoid false judgments caused by weak clutter interference. Second, the track associated with this label must have been stably output for at least a minimum number of consecutive frames. This ensures that the target is not a newly initiated false track, as transient clutter-induced components rarely maintain stable output over multiple frames.

As shown in Figure 4, when the dominant component’s weight drops below the threshold, and the track has met the stable output requirement, the algorithm confirms a missed alarm. The specific process is as follows:

To distinguish different targets, a label τ is introduced.

$T_{\tau }^{k-1}=\{\tau_{i}^{k-1}|i=1,2,\ldots ,L_{k-1}\}$ is the set of all labels of the targets output at time k−1. All components after the update at time k can be expressed as $\Psi (r_{k})=\{\omega_{k}^{i}(r_{k}),m_{k}^{i}(r_{k}),P_{k}^{i}(r_{k})){\}}_{i=1}^{J_{k}(r_{k})}$. For all $l\in \{1,2,\ldots ,L_{k-1}\}$, find Gaussian components bearing identical label to l−th track among all motion models at step k, and determine the component with the highest weight.

(28) $$i_{\max}=\underset{i\in 1:J_{k}^{l}}{\arg\max({\tilde{\omega }}_{k}^{i})},$$

where $J_{k}^{l}$ signifies the total number of Gaussian components across models with identical labels l, and ${\tilde{\omega }}_{k}^{i}$ represents the weight of the Gaussian component for that label at time k.

This implies that the measurement of the labeled target at time k may be lost after the measurement update at step k if ${\tilde{\omega }}_{k}^{i_{\max}}<TH_{\omega }$, and the track with this label has output at least $N_{\min}$ measurements at time k−1. Here, $TH_{\omega }$ is the missed-alarm threshold. Output the minimum number of measurements to ensure that the judged missed-alarm targets are all definite and stable. This method is not affected by clutter, so it will not increase the weights of non-targets, thus avoiding the generation of false targets.

#### 3.2.5. Missed Alarm Correction

After detecting a missed alarm, the missed alarm is corrected using the following equations:

(29) $$\omega_{k}^{A}(r_{k})=\eta \omega_{k|k-1}^{l}(r_{k}),$$

(30) $$m_{k}^{A}(r_{k})=m_{k|k-1}^{l}(r_{k}),P_{k}^{A}(r_{k})=P_{k|k-1}^{l}(r_{k}),$$

(31) $$\begin{array}{l} {\Psi^{\mathrm{new}}(r_{k})=\{\omega_{k}^{i}(r_{k}),m_{k}^{i}(r_{k}),P_{k}^{i}(r_{k})\}}_{i=1}^{J_{k}(r_{k})}\\ \;\;\;\;\;\;\;\;\;\;\;\;\;\;\;\;\cup \{\omega_{k}^{A}(r_{k}),m_{k}^{A}(r_{k}),P_{k}^{A}(r_{k})\}, \end{array}$$

(32) $$\eta =1-\frac{\left|\Delta v\right|}{v_{k-1}^{l}+v_{k-2}^{l}},$$

where η<1 denotes the weight penalty factor. $v_{k-1}^{l}$ and $v_{k-2}^{l}$ represent the Cartesian velocity magnitudes of the missed-alarm target at k−1 and k−2, respectively, and $\left|\Delta v\right|$ is the absolute value of the velocity change between the two previous time instants. As depicted in Figure 4, this figure details the entire missed-alarm correction process. The target missed alarm is corrected through the target prediction component, and the prediction components of all models for this target are added to the component set $\Psi^{\mathrm{new}}$. The specific process is shown in Algorithm 1.

The practical implementation involves setting $N_{\min}$ typically within 3–5 frames, with higher values recommended in dense clutter environments to mitigate false alarms. Concurrently, η should be configured between 0.3 and 0.9; elevated values are advised in scenarios prone to frequent missed detections to enhance trajectory continuity and reinforce tracking robustness.

Algorithmically, it is intuitively evident that the greater the variation in a target’s movement speed, the larger the penalty factor applied to correct missed alarms. This aligns with intuitive logic: when a target’s motion state changes frequently and involves large-scale maneuvers, our confidence in estimating its next state diminishes. Conversely, when a target maintains a stable movement pattern, we can rely on a specific motion model for prediction.

**Algorithm 1**. The Algorithm for Judgment and Correction of Missed Alarm**Input **$T_{\tau }^{k-1}=\{\tau_{i}^{k-1}|i=1,2,\ldots ,L_{k-1}\}$, $\Psi (r_{k})=\{\omega_{k}^{i}(r_{k}),m_{k}^{i}(r_{k}),P_{k}^{i}(r_{k})){\}}_{i=1}^{J_{k}(r_{k})}$, weight penalty factor η and the missed-alarm threshold $TH_{\omega }$

**For** $l\in \{1,2,\ldots ,L_{k-1}\}$

     $i_{\max}=\underset{i\in 1:J_{k}^{l}}{\arg\max({\tilde{\omega }}_{k}^{i})}$,

3.       **If**  ${\tilde{\omega }}_{k}^{i_{\max}}<TH_{\omega }$4.            $\eta =1-\frac{\left|\Delta v\right|}{v_{k-1}^{l}+v_{k-2}^{l}}$,5.            $m_{k}^{A}(r_{k})=m_{k|k-1}^{l}(r_{k}),P_{k}^{A}(r_{k})=P_{k|k-1}^{l}(r_{k})$,6.            $\omega_{k}^{A}(r_{k})=\eta \omega_{k|k-1}^{l}(r_{k})$,

7.            $\begin{array}{l} \Psi^{\mathrm{new}}(r_{k})={\left\{\omega_{k}^{i}(r_{k}),m_{k}^{i}(r_{k}),P_{k}^{i}(r_{k})\right\}}_{i=1}^{J_{k}(r_{k})}\\ \;\;\;\;\;\;\;\;\;\;\;\;\;\;\;\;\cup \{\omega_{k}^{A}(r_{k}),m_{k}^{A}(r_{k}),P_{k}^{A}(r_{k})\}, \end{array}$,8.      **End**9.
**End**

**Output **

$\Psi^{\mathrm{new}}(r_{k})$



#### 3.2.6. Pruning and Absorption

During this process, similar components are combined into one.

Herein, the Mahalanobis distance measures the distance between hybrid components; small-weight components within the absorption threshold are merged with large-weight ones. Notably, the absorption threshold is adaptive, related to the weights of components to be absorbed and measurement noise, defined as

(33) $$Th_{\mathrm{GM}}^{i}=(1+\omega_{k}^{i}(r_{k}))\mathrm{tr}(R_{r_{k}}^{k}),$$

where $Th_{\mathrm{GM}}^{i}$ denotes the absorption threshold, $\omega_{k}^{i}(r_{k})$ denotes the weight under model $r_{k}$ of the i−th component to be absorbed, and $\mathrm{tr}(R_{r_{k}}^{k})$ denotes the trace of the measurement noise matrix.

For real targets, their Gaussian components maintain high weights due to distinct RCS and Doppler features that match target characteristics, leading to a larger merging threshold. This reduces the risk of erroneous merging of real target components.

In contrast, clutter or false targets, with features mismatched to real targets, have low component weights, resulting in a smaller merging threshold. These low-weight components are either absorbed by high-weight, real target components or pruned during processing, eventually being eliminated. This method can effectively identify the similarity of different hybrid components. The specific pruning and absorption procedure is outlined in Algorithm 2.

For the specific component merging strategy, we employ an approach where higher-weight components absorb similar ones. After absorption, the state of the absorbed components is directly determined by the high-weight component, rather than through weighted fusion of all merged components. This aligns with the earlier feature matching process. By reducing clutter interference, direct use of high-weight components further improves the accuracy of the merged state while reducing computation time throughout the merging process.

**Algorithm 2.** The Algorithm for Pruning and Absorption**Input **${\left\{\{\omega_{k}^{i}(r_{k}),m_{k}^{i}(r_{k}),P_{k}^{i}(r_{k}))\right\}}_{i=1}^{J_{k}(r_{k})}{\}}_{r_{k}=1}^{n_{r}}$, a pruning threshold $\Gamma_{p}$, a absorption threshold ThGM, maximum Gaussian terms $J_{\max}$ and $M=\{i=1,\ldots ,J_{k}(r_{k})|(\omega_{k}^{i}(r_{k})>T_{p})\}$** Set **L = 0.
**Loop**

1.l=l+1.

2.$j=\arg\max\omega_{k}^{i}(r_{k})$.3.

$Th_{\mathrm{GM}}^{i}=(1+\omega_{k}^{i}(r_{k}))\sigma_{s}$

4.

$L=\{i\in Μ:{\left(m_{k}^{i}(r_{k})-m_{k}^{j}(r_{k})\right)}^{T}{\left(P_{k}^{i}(r_{k})\right)}^{-1}(m_{k}^{i}(r_{k})-m_{k}^{j}(r_{k}))<Th_{\mathrm{GM}}^{i}\}.$

5.${\bar{\omega }}_{l}^{k}(r_{k})={\sum^{n}}_{i\in L}\omega_{k}^{i}(r_{k})$,

6.$\Omega_{k}^{l}(r_{k})=\{{\bar{\omega }}_{k}^{l}(r_{k}),m_{k}^{j}(r_{k}),P_{k}^{j}(r_{k})\}$,c7.

Μ=Μ\L.

8.**If **(Μ=∅), **break.**9.**If **$l>J_{\max}$, remove the Gaussian components in ascending order of their weights until the number of Gaussian components is less than or equal to $J_{\max}$.
**Output **

${\left\{\Omega_{k}^{j}(r_{k})\right\}}_{j=1}^{\min\{l,J_{\max}\}}.$



## 4. Simulation

In this section, we evaluate the performance of the proposed algorithm. The tracking capability of the FM-MM-GM-PHD filter for multiple maneuvering targets is assessed under both uniform and non-uniform clutter scenarios. Experiments demonstrate that this method can precisely monitor multiple maneuvering targets and determine the exact number of targets in both clutter environments. Herein, the Optimal Sub-Pattern Assignment (OSPA) distance is employed to quantify the algorithm’s tracking performance, defined as

(34) $${\bar{d}}_{p}^{c}(X,Y)={\left\{n^{-1}\left[\underset{ο\in Q_{n}}{\min}\sum_{i=1}^{n}{\left(d^{\left(c\right)}(x_{i},y_{ο(i)})\right)}^{p}+c^{p}(n-m)\right]\right\}}^{\frac{1}{p}},$$

where ${\bar{d}}_{p}^{c}(X,Y)$ denotes the set-to-set distance between X and Y. The parameter p regulates the sensitivity to track loss, and the cut-off parameter c specifies penalty for positioning and cardinality estimation errors. In the experiments of this paper, the above parameter is set to p=2, c=200. p=2 deliberately intensifies sensitivity to target maneuvering by quadratically amplifying state estimation errors during kinematic transitions. Meanwhile, c=200 serves dual purposes: it aligns with the spatial resolution of passive radar systems while preventing premature truncation of localization deviations, thereby preserving metric fidelity to true tracking errors.

The RCS–Doppler-assisted feature-matching mechanism extracts two discriminative target characteristics to augment measurement association. RCS values are obtained through logarithmic conversion of the received-to-transmitted power ratio, incorporating bistatic range and antenna gain parameters. Simultaneously, Doppler frequency is derived from radial velocity measurements relative to the receiver.

In the simulation, one hundred independent Monte Carlo runs were conducted, with each experiment spanning a duration of 100 s and the sampling time interval being T=1 s. The standard deviation of the bistatic range noise $\sigma_{R}=10\;m,$ the standard deviation of the bistatic velocity noise $\sigma_{v}=2\;m/s,$ and the standard deviation of the angular noise $\sigma_{a}=1{}^{\circ}$. The clutter is Poisson-distributed in a two-dimensional space, and the RCS likelihood function of the clutter satisfies the Rayleigh distribution. In the two-dimensional simulation scenario, four targets are set to have multiple maneuvers in space, with a total duration of 100 s. The target parameters are provided in Table 1.

The parameters of the birth target model are given by

$$m_{\gamma }^{1}={\left[10000,120,10000,40\right]}^{T},$$

$$m_{\gamma }^{2}={\left[-2000,30,2000,100\right]}^{T},$$

$$m_{\gamma }^{3}={\left[4000,-100,3000,30\right]}^{T},$$

$$P_{\gamma }=diag({\left[1\;0.01\;1\;0.01\right]}^{T}).$$

**Figure 5:**
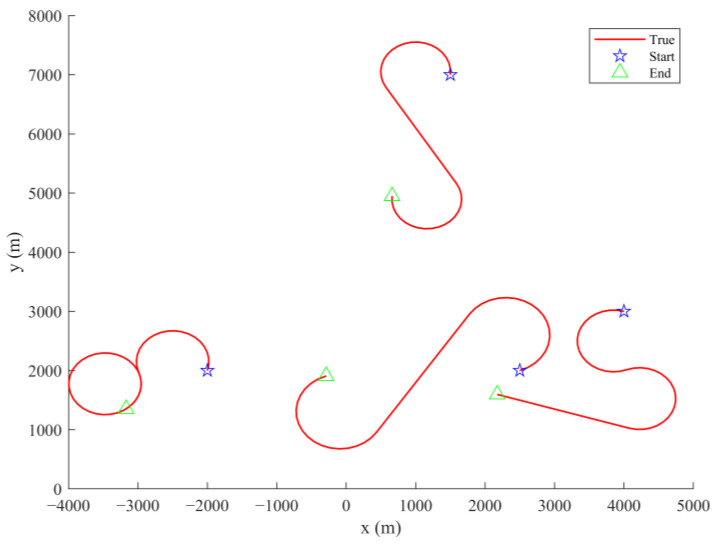
The true tracks of the targets.

The pruning threshold is set to $\Gamma_{p}\;=\;10^{-5}$ in order to filter out negligible low-weight components, and the maximum value of components is limited to $J_{max}\;=\;100$ for constraining computational complexity while supporting multi-target tracking. The target trajectory is given in Figure 5.

### 4.1. Scenario 1

**Figure 6:**
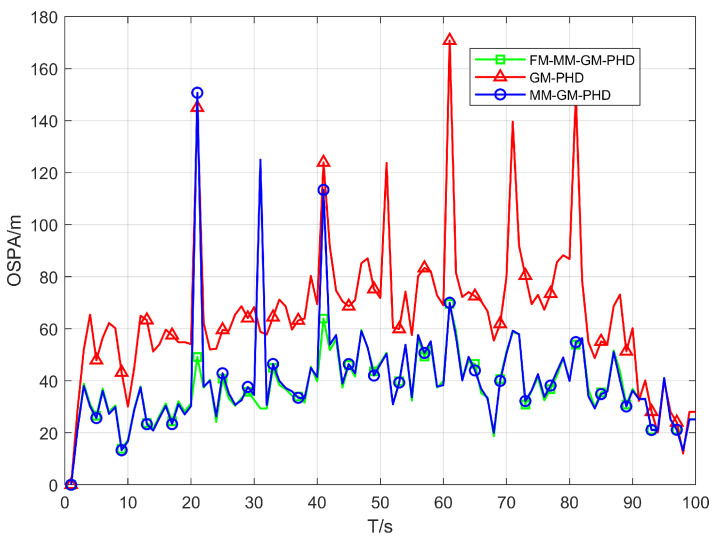
OSPA error of three filters in uniform clutter (Pd=0.9).

**Figure 7:**
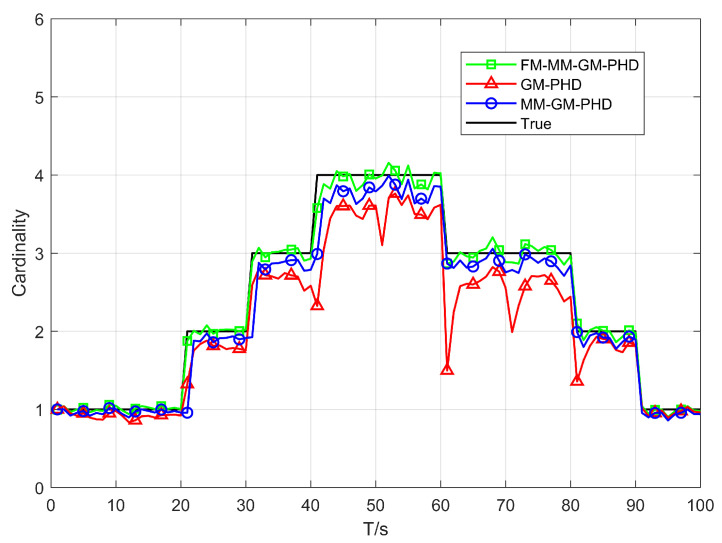
Cardinality estimates of three filters in uniform clutter (Pd=0.9).

In Scenario 1, we conduct a performance test in a uniform clutter scenario with the number of clutter per frame r=50 and a detection probability Pd=0.9. We benchmark the performance of the conventional GM-PHD, MM-GM-PHD, and our proposed FM-MM-GM-PHD filter. The tracking and cardinality estimation accuracies of the three methods are illustrated in Figure 6 and Figure 7. In uniform clutter scenario, the OSPA distance curve of the FM-MM-GM-PHD filter is very close to the MM-GM-PHD filter, and both are significantly better than the GM-PHD filter. This is because the multi-model approach can handle the maneuvers of multiple targets. However, the MM-GM-PHD filter has obvious spikes at some moments, while the proposed algorithm does not. In terms of cardinality estimation, the MM-GM-PHD and GM-PHD both underestimate target numbers. The latter suffers from severe underestimation due to target maneuvers. By contrast, the FM-MM-GM-PHD filter accurately estimates target cardinality thanks to its missed-alarm correction mechanism. Moreover, the method we propose can rapidly pinpoint the emergence and vanishing of targets.

**Figure 8:**
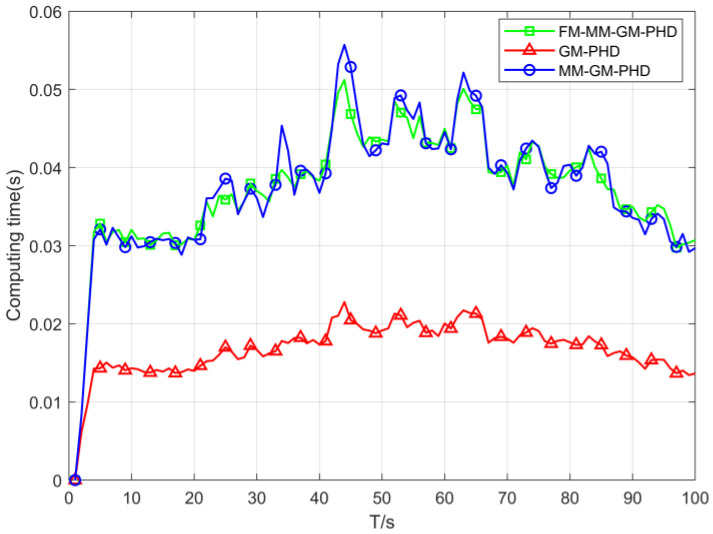
Computing time of three filters (Pd=0.9).

Figure 8 illustrates the computational time of three filters within a uniform clutter environment. As evident from the results, the computational complexity of the proposed algorithm aligns with that of the MM-GM-PHD, while both are more computationally intensive than the GM-PHD.

**Figure 9:**
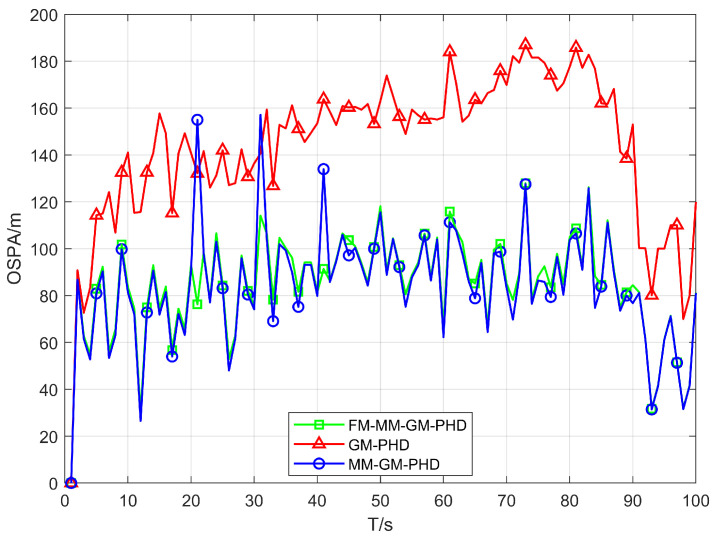
OSPA error of three filters in uniform clutter (Pd=0.7).

**Figure 10:**
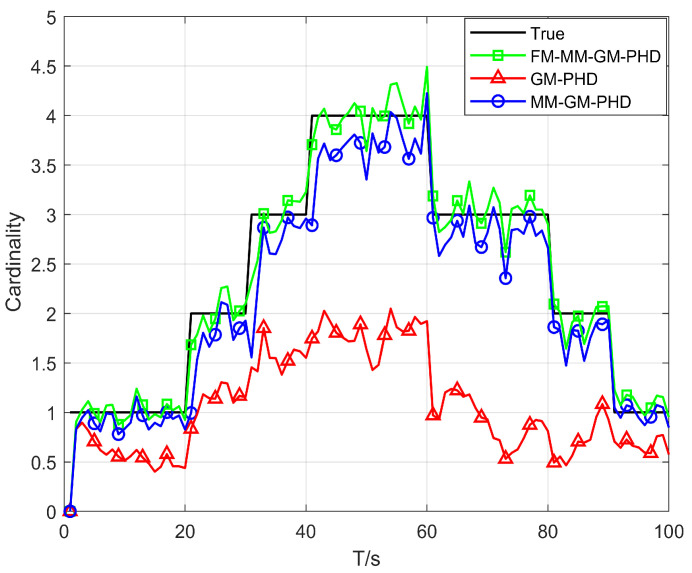
Cardinality estimates of three filters in uniform clutter (Pd=0.7).

To test the performance of the proposed missed alarm mechanism in a uniform scenario, we compare the proposed algorithm with other filters under low detection probability. Figure 9 and Figure 10 show the OSPA and cardinality estimation results under the condition of a detection probability of 0.7, respectively. When the detection probability is low, GM-PHD can hardly maintain continuous tracking and significantly underestimates the number of targets. The proposed FM-MM-GM-PHD algorithm still remains comparable to MM-GM-PHD in terms of OSPA, but the latter more severely underestimates the targets in cardinality estimation.

Through its missed-detection correction mechanism, the proposed algorithm generally attains accurate target number estimation under low detection probabilities, with negligible impact on the OSPA distance.

### 4.2. Scenario 2

**Figure 11:**
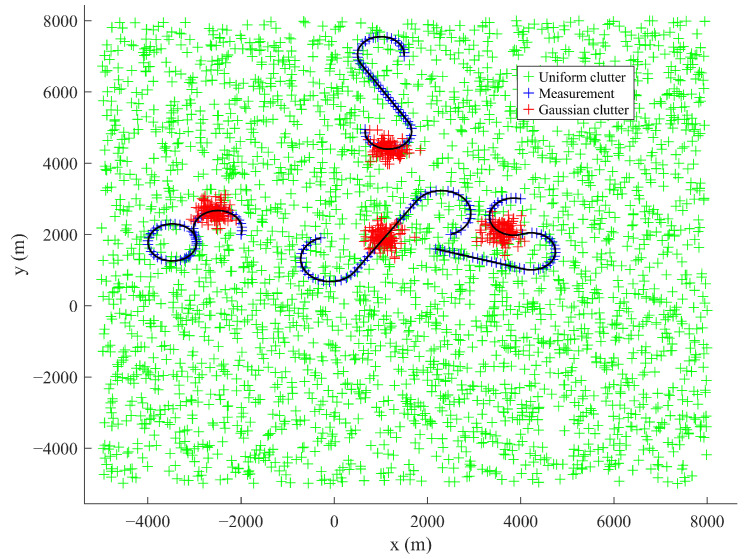
Measurements in non-uniform clutter.

**Figure 12:**
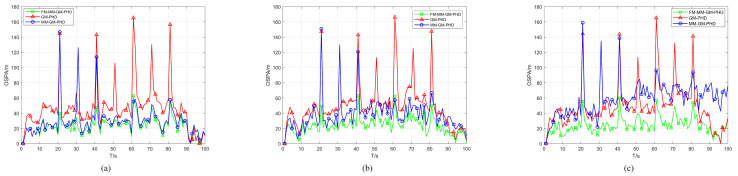
OSPA error of three filters in different non-uniform clutter. (**a**) rr=5. (**b**) rr=10. (**c**) rr=20.

**Figure 13:**
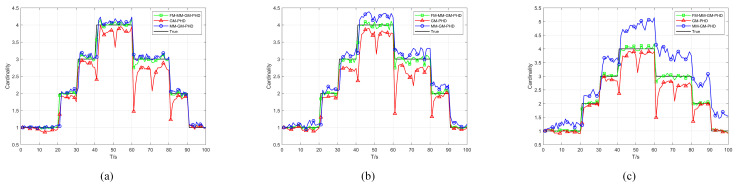
Cardinality estimates of three filters in different non-uniform clutter. (**a**) rr=5. (**b**) rr=10. (**c**) rr=20.

Scenario 2 benchmarks the proposed algorithm against GM-PHD and MM-GM-PHD in a non-uniform clutter scenario. As Figure 11 illustrates, the scenario involves uniform clutter combined with dense Gaussian-distributed clutter, where the central position of the Gaussian non-uniform clutter is randomly located on each target trajectory. The detection probability is fixed at Pd=0.98. Across different experiments, the number of non-uniform clutter rr in each area per frame was set to 5, 10, and 20, respectively, for each trial. Figure 12 and Figure 13 show the average OSPA distances for different algorithms under different non-uniform clutter conditions. It indicates that when the number of non-uniform clutter per frame in each area rr=5, FM-MM -GM-PHD slightly better than MM-GM-PHD while the OSPA of the GM-PHD filter is much higher than the other methods, which is caused by target maneuvers. In terms of cardinality estimation, FM-MM-GM-PHD has the most accurate cardinality estimation ability and can detect the appearance of new targets first. As the number of non-uniform clutter increases, the OSPA of MM-GM-PHD rises rapidly, and the cardinality estimation is significantly overestimated. This occurs because the rise in dense clutter within the multi-model gate causes the creation of false targets. In contrast, FM-MM-GM-PHD always maintains good performance.

**Figure 14:**
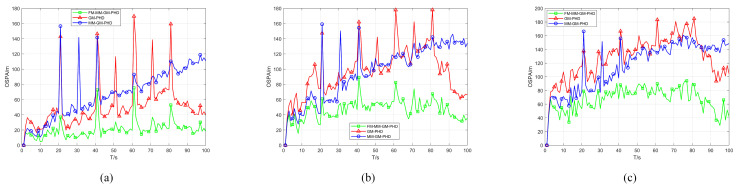
OSPA error of three filters in non-uniform clutter with different probabilities of detection. (**a**) Pd=0.98. (**b**) Pd=0.88. (**c**) Pd=0.78.

**Figure 15:**
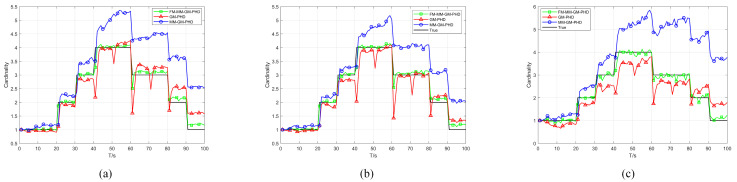
Cardinality estimates of three filters in non-uniform clutter with different probabilities of detection. (**a**) Pd=0.98. (**b**) Pd=0.88. (**c**) Pd=0.78.

Figure 14 and Figure 15 illustrate the performance of the proposed algorithm and comparative methods in a non-uniform clutter under different detection probabilities. With a fixed number of uniform clutter per frame r=50 and non-uniform Gaussian-distributed clutter per frame rr=20 in each area, the detection probabilities Pd are set as 0.98, 0.88, and 0.78, respectively. As illustrated in Figure 14, we compared the OSPA distances of different algorithms with declining detection probability. It indicates that as the detection probability decreases, the OSPA distances of all algorithms increase. However, the FM-MM-GM-PHD filter is affected the least and still has a relatively small OSPA distance compared with other filters. This is because the missed-alarm correction mechanism of the proposed method can effectively deal with target missed alarms.

Figure 15 shows the estimation of the cardinality of targets by all filters under different detection probabilities. The FM-MM-GM-PHD filter still has the highest estimation accuracy compared with other filters and is quite close to the true value of the cardinality of targets. As the detection probability decreases, the performance of other filters in estimating the cardinality of targets drops sharply. The MM-GM-PHD filter severely overestimates the number of targets due to the multi-model approach.

However, our experiments have also identified specific edge cases worth noting. Specifically, when targets exhibit crossing trajectories and share highly similar RCS and Doppler features, the current feature-matching framework shows reduced discriminability. In such scenarios, inadequate adjustment of features can lead to false association between measurements and targets, resulting in overestimation or underestimation of target cardinality.

**Figure 16:**
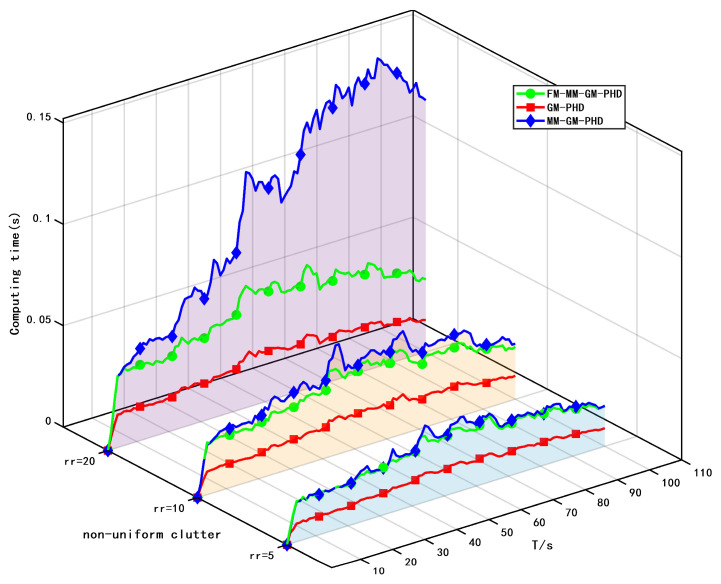
Computing time under different non-uniform clutter conditions.

Figure 16 also illustrates the real-time computation times of different filters under varying non-uniform clutter conditions. In all scenarios, the GM-PHD filter has the shortest computation time because it does not need to adopt a multi-model approach to consider targets. When the non-uniform clutter count is 5, the proposed algorithm is slightly more computationally efficient than MM-GM-PHD. As the non-uniform clutter count increases, the computation time of the proposed algorithm becomes significantly shorter. This is because the proposed algorithm suppresses false targets via target features, thereby reducing unnecessary estimation of false targets. In contrast, as dense non-uniform clutter increases, the traditional MM-GM-PHD—unable to distinguish true from false targets—processes a large number of components simultaneously through PHD filtering, greatly increasing computation time.

**Figure 17:**
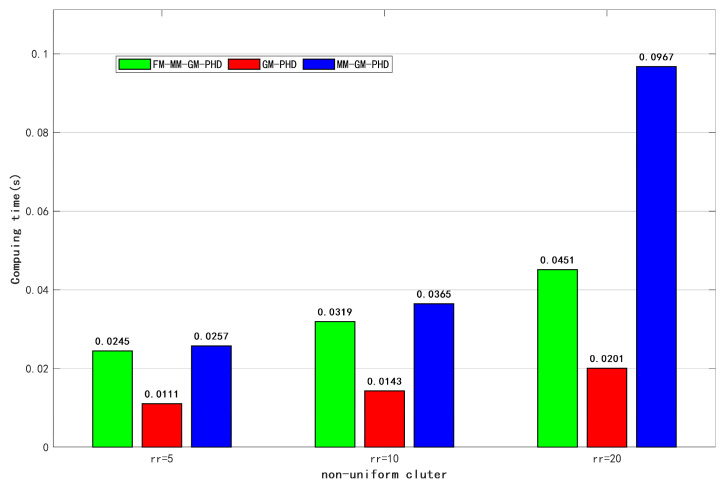
Average computing time for different non-uniform clutter.

Figure 17 presents the comparative computational efficiency of three distinct filters under escalating non-uniform clutter conditions. In the non-uniform clutter conditions, the proposed algorithm demonstrates progressively superior computational performance, particularly evident when rr=20. Crucially, the results confirm that our approach maintains stable processing times even at peak clutter achieving up to 60% reduction compared to MM-GM-PHD. Collectively, these computational advantages complement the method’s established tracking accuracy and cardinality estimation superiority in non-uniform clutter environments.

### 4.3. Computational Complexity Analysis

The computational complexity of the MM-GM-PHD and proposed FM-MM-GM-PHD filters is quantified using floating-point operations (FLOPs) as the metric, following the approach in. For the MM-GM-PHD filter, its complexity arises primarily from multi-model operations; each of the M motion models independently performs prediction and update steps, contributing $O(M\cdot N_{a}\cdot (d_{s}^{2}+d_{m}\cdot d_{s}))$, where $d_{s}$ is the state dimension, $d_{m}$ is the measurement dimension, and $\;N_{a}$ is the number of Gaussian components. Additional overhead comes from model probability fusion $\left(O(M^{2}\cdot N_{a})\right)$ and scaled pruning and merging operations due to the increased number of components across models $(O(M^{2}\cdot N_{a}^{2})).$ Consequently, the total complexity of MM-GM-PHD is $O(M\cdot N_{a}\cdot (d_{s}^{2}+d_{m}\cdot d_{s})+M^{2}\cdot N_{a}+M^{2}\cdot N_{a}^{2}$, which grows significantly with non-uniform clutter intensity as $N_{a}$ increases due to false measurements. In contrast, the FM-MM-GM-PHD filter introduces two key optimizations to reduce complexity while maintaining performance. First, feature matching (using RCS and Doppler features, f=2) adds a negligible overhead of $O(N_{a}\cdot f)$. Second, adaptive pruning suppresses false components. Both reduce the effective number of Gaussian components to $\alpha \cdot N_{a}$, where α≪1. The missed-alarm correction mechanism contributes a minor cost of $O(N_{t})$ Thus, the total complexity of FM-MM-GM-PHD is $O(\alpha \cdot M\cdot N_{a}\cdot (d_{s}^{2}+d_{m}\cdot d_{s}+f)+\alpha^{2}\cdot M^{2}\cdot N_{a}^{2}+N_{t})$, and the memory requirement is $O(\alpha \cdot M\cdot N_{a}\cdot (d_{s}^{2}+d_{s})+M\cdot d_{s}+N_{t}\cdot d_{s}).$ Meanwhile, the actual computational time of the algorithm is not only related to model complexity but also to many other factors, such as memory access cost, hardware characteristics, software implementation, and system environment. Model complexity cannot accurately measure the estimation speed of the proposed algorithm. However, the simulation experiments in this paper further well demonstrate the advantages of the proposed algorithm in non-uniform clutter environments. The reduction in effective components via α makes FM-MM-GM-PHD significantly more efficient than MM-GM-PHD, with computational time decreasing by 60% in dense non-uniform clutter.

### 4.4. Results Discussion

As the amount of non-uniform clutter increases, the conventional MM-GM-PHD algorithm will significantly overestimate the number of targets, resulting in a large number of false targets. This issue not only substantially increases the computational load of subsequent filtering processes but also causes a great deal of unnecessary estimation resources to be wasted on false targets. In contrast, the method proposed in this paper can effectively mitigate the negative impacts of non-uniform clutter on multi-model approaches, enabling accurate estimation of the number and states of multiple maneuvering targets. Regarding computational complexity, the proposed method is comparable to MM-GM-PHD in non-uniform clutter environments. However, as the number of non-uniform clutter further increases, the computational time advantage of FM-MM-GM-PHD becomes increasingly prominent.

Quantitative analysis confirms the FM-MM-GM-PHD filter’s significant advantages over conventional MM-GM-PHD in non-uniform clutter. Under rr=20, the proposed method reduces cardinality estimation error by about 90% and lowers OSPA distance by about 75%. This stems from RCS–Doppler feature matching, which suppresses approximately 97% of clutter-induced false targets through joint likelihood ratios, while the missed-alarm correction mechanism maintains cardinality deviations below 5% when the detection probability is low. Computational efficiency improves by approximately 60% at high clutter loads due to reduced false component processing. The key limitations involve moderate precision degradation during extreme non-linear maneuvers such as high-g turns, alongside reliance on measurable RCS and Doppler features. Future work will integrate particle filtering to enhance non-linear tracking capability in passive radar bistatic geometries, particularly for swarm targets with interactive motions.

## 5. Conclusions

This paper proposes an RCS–Doppler-assisted multi-model PHD filter for passive radar. It suppresses false targets in non-uniform clutter by adaptively weighting Gaussian components using radar echo features. Moreover, the missed-detection correction mechanism integrated into the proposed algorithm compensates for measurement loss using targets’ historical kinematic states. Simulation results demonstrate that the proposed algorithm significantly outperforms traditional MM-GM-PHD filter and GM-PHD filter in both uniform and non-uniform clutter scenarios. Specifically, in non-uniform environments with dense Gaussian clutter, its OSPA error is approximately 30% lower than that of traditional algorithms, and it is particularly accurate in estimating target cardinality. As detection probability continues to decrease, the proposed algorithm also exhibits stronger robustness in both OSPA and cardinality estimation compared to other methods.

Future work will focus on developing interaction-aware motion models to enhance tracking performance in target-congested scenarios, with applications for swarm tracking.

## Figures and Tables

**Table 1:** The parameters of the targets in the experiment.

Target	State (m,m/s,m,m/s)	Birth (s)	Death (s)
**1**	$\left[1500,10,7000,100\right]$	1	60
**2**	$\left[10000,120,10000,40\right]$	20	80
**3**	$\left[-2000,30,2000,100\right]$	40	100
**4**	$\left[4000,-100,3000,30\right]$	30	90

## Data Availability

The original contributions presented in this study are included in the article. Further inquiries can be directed to the corresponding author.
